# Preclinical Evaluation of Biodistribution and Toxicity of [^211^At]PSMA-5 in Mice and Primates for the Targeted Alpha Therapy against Prostate Cancer

**DOI:** 10.3390/ijms25115667

**Published:** 2024-05-23

**Authors:** Tadashi Watabe, Kazuko Kaneda-Nakashima, Yuichiro Kadonaga, Kazuhiro Ooe, Thosapol Sampunta, Naoki Hirose, Xiaojie Yin, Hiromitsu Haba, Yukiyoshi Kon, Atsushi Toyoshima, Jens Cardinale, Frederik L. Giesel, Koichi Fukase, Noriyuki Tomiyama, Yoshifumi Shirakami

**Affiliations:** 1 Department of Radiology, Graduate School of Medicine, Osaka University, Osaka 565-0871, Japan; 2 Institute for Radiation Sciences, Osaka University, Osaka 565-0871, Japan; 3 Core for Medicine and Science Collaborative Research and Education, Project Research Center for Fundamental Sciences, Graduate School of Science, Osaka University, Osaka 560-0043, Japan; 4 Institute of Experimental Animal Sciences, Faculty of Medicine, Osaka University, Osaka 565-0871, Japan; 5 Nishina Center for Accelerator-Based Science, RIKEN, Wako 351-0198, Japan; 6 Department of Nuclear Medicine, Medical Faculty and University Hospital Duesseldorf, Heinrich-Heine-University Duesseldorf, 40225 Duesseldorf, Germany; 7 Department of Chemistry, Graduate School of Science, Osaka University, Osaka 560-0043, Japan

**Keywords:** astatine, PSMA, prostate cancer, toxicity study, clinical trial

## Abstract

Astatine (^211^At) is a cyclotron-produced alpha emitter with a physical half-life of 7.2 h. In our previous study, the ^211^At-labeled prostate-specific membrane antigen (PSMA) compound ([^211^At]PSMA-5) exhibited excellent tumor growth suppression in a xenograft model. We conducted preclinical biodistribution and toxicity studies for the first-in-human clinical trial. [^211^At]PSMA-5 was administered to both normal male ICR mice (*n* = 85) and cynomolgus monkeys (*n* = 2). The mice were divided into four groups for the toxicity study: 5 MBq/kg, 12 MBq/kg, 35 MBq/kg, and vehicle control, with follow-ups at 1 day (*n* = 10 per group) and 14 days (*n* = 5 per group). Monkeys were observed 24 h post-administration of [^211^At]PSMA-5 (9 MBq/kg). Blood tests and histopathological examinations were performed at the end of the observation period. Blood tests in mice indicated no significant myelosuppression or renal dysfunction. However, the monkeys displayed mild leukopenia 24 h post-administration. Despite the high accumulation in the kidneys and thyroid, histological analysis revealed no abnormalities. On day 1, dose-dependent single-cell necrosis/apoptosis was observed in the salivary glands of mice and intestinal tracts of both mice and monkeys. Additionally, tingible body macrophages in the spleen and lymph nodes indicated phagocytosis of apoptotic B lymphocytes. Cortical lymphopenia (2/10) in the thymus and a decrease in the bone marrow cells (9/10) were observed in the 35 MBq/kg group in mice. These changes were transient, with no irreversible toxicity observed in mice 14 days post-administration. This study identified no severe toxicities associated with [^211^At]PSMA-5, highlighting its potential as a next-generation targeted alpha therapy for prostate cancer. The sustainable production of ^211^At using a cyclotron supports its applicability for clinical use.

## 1. Introduction

Prostate-specific membrane antigen (PSMA) is a membrane protein highly expressed in most prostate cancers [1,2,3]. Radioligand therapy that targets PSMA has emerged as an effective treatment option for refractory prostate cancer. [^177^Lu]PSMA-617 has been approved for metastatic castration-resistant prostate cancer (mCRPC) treatment in the United States and Europe [4,5,6]. It has significantly extended overall survival in patients with mCRPC previously treated with androgen receptor pathway inhibitors (ARPI) and taxane therapy compared to the standard of care in the VISION trial [7]. Additionally, [^177^Lu]PSMA-617 has been shown to prolong radiographic progression-free survival in taxane-naïve patients with mCRPC compared to ARPI changes, as reported in the PSMAfore trial [8]. However, some patients remain refractory or exhibit early recurrence after [^177^Lu]PSMA-617 therapy. Alpha therapy using [^225^Ac]PSMA-617 has proven effective in patients resistant to beta therapy with [^177^Lu]PSMA-617 [9,10,11,12,13,14,15,16]. A Phase 1/2 clinical trial employing [^225^Ac]PSMA-617 and other ^225^Ac-labeled PSMA-targeted compounds is currently underway. However, the global supply of ^225^Ac is constrained by production limitations that require nuclear fuel materials ([^229^Th] or [^232^Th]) or rare radioisotopes ([^226^Ra]) [17]. Recently, there has been increasing interest in alternative alpha emitters beyond ^225^Ac. Astatine (^211^At), an alpha emitter with a physical half-life of 7.2 h, can be produced using a 30 MeV ^4^He cyclotron with a natural bismuth (^209^Bi) target [18,19,20,21,22]. Given this target material’s natural abundance, ^211^At can be produced globally by establishing a manufacturing base equipped with a cyclotron that enables a stable and sustainable supply. Moreover, the simple decay chain of ^211^At offers advantages by eliminating the need for long-term radioactivity management and concerns related to the redistribution of daughter nuclides in the body.

We developed a novel ^211^At-labeled PSMA compound ([^211^At]PSMA-5) that showed high therapeutic efficacy in a mouse xenograft model of prostate cancer [23]. Our goal was to start an investigator-initiated, first-in-human clinical trial for patients with mCRPC. In preparation, we consulted the Pharmaceuticals and Medical Devices Agency (PMDA) of Japan and reached an agreement on the preclinical safety study requirements in alignment with the International Council for Harmonization (ICH) guidelines (M3 and S9) and a previous study using [^211^At]NaAt [24].

In this study, we conducted biodistribution and extended single-dose toxicity studies of [^211^At]PSMA-5 in rodents and monkeys, adhering to the reliability standards of pharmaceutical law. This approach is essential to determine the starting dose in Phase-1 clinical trial (first-in-human).

## 2. Results

**Figure 1:**
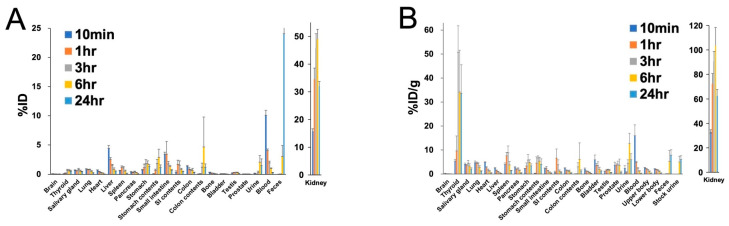
Biodistribution ((**A**) %ID and (**B**) %ID/g) in ICR mice at 10 min, 1 h, 3 h, 6 h, and 24 h post-administration of [^211^At]PSMA-5.

The biodistribution in ICR mice is shown in Figure 1. The kidneys exhibited the highest uptake, peaking at 6 h post-administration, reflecting physiological PSMA expression in the proximal tubules. A relatively high uptake was also observed in the thyroid gland, reaching its peak after 3 h, suggesting a minor release of free At^−^ from the compound. The cumulative urinary excretion was 4.85 ± 1.76 %ID at 6 h and 18.89 ± 4.57 %ID at 24 h, while the cumulative fecal excretion was 3.03 ± 1.90 %ID at 6 h and 24.07 ± 8.54 %ID at 24 h.

**Figure 2:**
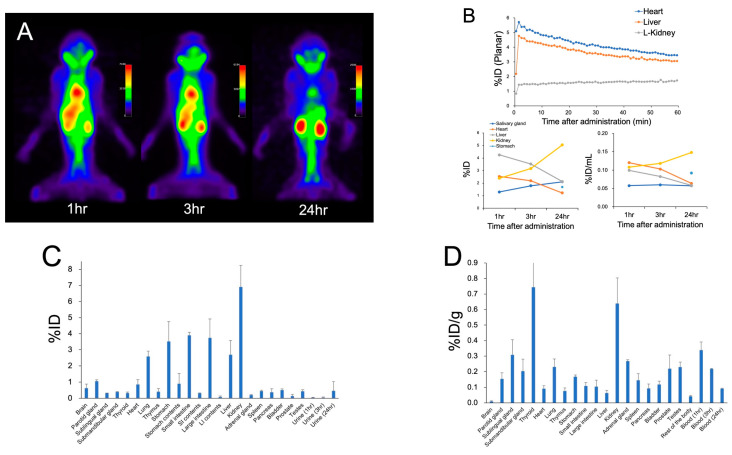
(**A**) SPECT images (maximum intensity projection) of the monkey after administration of [^211^At]PSMA-5. (**B**) Time–activity curves from the ROI analysis of planar and SPECT images. Biodistribution ((**C**) %ID and (**D**) %ID/g) in cynomolgus monkey 24 h after administration of [^211^At]PSMA-5.

In the cynomolgus monkey, planar images revealed accumulation in the heart and liver, mirroring blood pool radioactivity, up to 3 h post-administration (Figure 2A,B). A progressive increase in physiological accumulation was observed in the kidneys. In contrast, urinary excretion was not detected in the bladder. A mild accumulation in the salivary glands was evident 1 h post-administration. At the 24 h autopsy, relatively high accumulation was observed in both the kidneys and thyroid, paralleling the biodistribution observed in mice (Figure 2C,D). Radioactivity in the blood decreased over time, and the main excretion route was presumed to be through feces via the gastrointestinal tract. The cumulative urinary and fecal excretions at 24 h were 0.46 %ID and 11.27 %ID, respectively.

In the TLC analysis of the blood samples, [^211^At]PSMA-5 was solely present in both mice and cynomolgus monkeys, and no metabolites were detected.

The highest estimated absorbed dose in humans was recorded for the kidneys at 4.05 mGy/MBq (Table 1). Subsequent doses were observed in the thyroid at 1.82 mGy/MBq, stomach at 0.258 mGy/MBq, spleen at 0.242 mGy/MBq, cardiac muscle at 0.232 mGy/MBq, and salivary glands at 0.218 mGy/MBq. For other organs such as the prostate, bladder, pancreas, brain, lungs, red bone marrow, liver, small intestine, colon, and testes, lower values were observed, ranging from 0.01 to 0.11 mGy/MBq.

**Figure 3:**
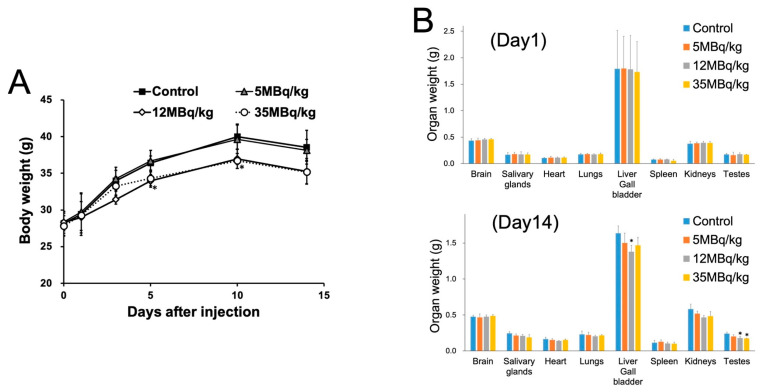
(**A**) Changes in body weight after administration of [^211^At]PSMA-5. (Note that the last measurement on day 14 was affected by fasting overnight for the blood examination.) (**B**) Comparison of organ weight after administration of [^211^At]PSMA-5 on days 1 and 14 (*: *p* < 0.05 compared with the control group using Dunnett’s test).

In the mice groups treated with 12 MBq/kg and the 35 MBq/kg, a significant decrease in body weight was observed on day 5 and day 10, respectively, compared to the control (Figure 3A), possibly due to the decrease in oral food intake. In the organ weight measurement of mice, a significant decrease in testes weight was observed in the 12 MBq/kg (organ weight: 0.18 ± 0.02 g) and 35 MBq/kg (organ weight: 0.17 ± 0.003 g) groups, compared to the control group (0.24 ± 0.02 g) on day 14.

In the blood examination of mice, no significant difference was observed between the 5, 12, and 35 MBq/kg [^211^At]PSMA-5 and control groups, either on day 1 or day 14, after administration (Table 2 and Supplemental Table S2), indicating no apparent myelosuppression or renal dysfunction. In cynomolgus monkeys, mild but significant leukopenia (mainly lymphopenia) was observed 24 h after administration, suggesting myelosuppression (Table 3). No significant changes were observed in renal function or electrolyte levels (Supplemental Table S3). At 24 h post-administration, one monkey (1/2) showed a mild increase in amylase, which might reflect mild injury to the salivary glands, although no abnormal findings were found in the histological examination described below. Elevated levels of AST, ALT, and CK were also observed in only one of the monkeys, possibly due to the hemolysis or unknown mechanism. LDH levels were elevated in both monkeys, although the reason remains uncertain.

**Figure 4:**
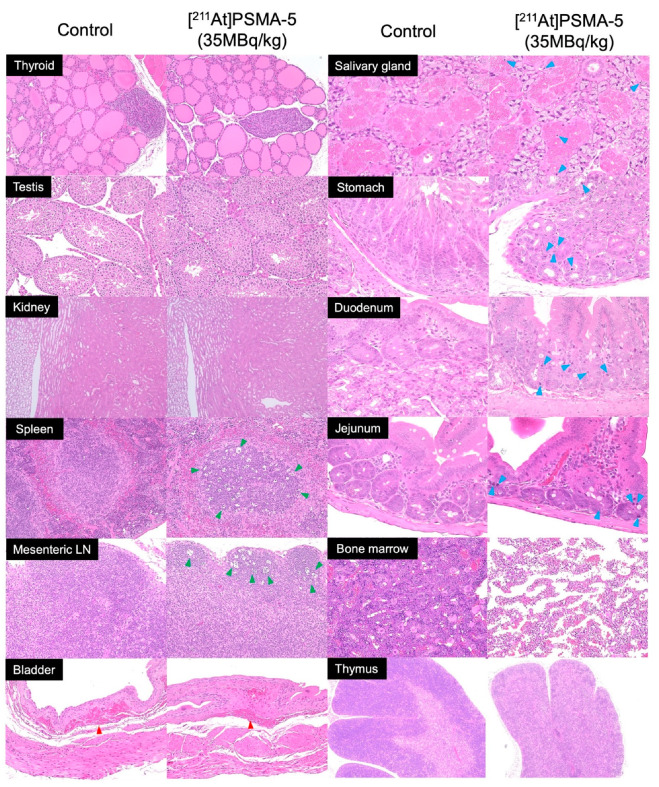
HE-stained section of ICR mice (day 1) after administration of [^211^At]PSMA-5 (35 MBq/kg) (magnification ×400). Single-cell necrosis/apoptosis of crypt epithelial cells (blue arrow heads) are observed in the salivary gland, stomach, duodenum, and jejunum. Tingible body macrophages (green arrow heads) were observed in the spleen and mesenteric lymph node (LN). Submucosal hemorrhages (red arrow heads) are observed in the bladder, both in the control and [^211^At]PSMA-5 (35 MBq/kg) groups, possibly due to the urine collection procedure. A decrease in bone marrow cells and a decrease in cortical lymphocytes in the thymus are observed.

During necropsy, expert evaluation revealed no gross abnormalities beyond spontaneous lesions (Supplemental Table S4). Histological results of the mice are shown in Figure 4 and Supplemental Tables S5-1 and S5-2. There were no significant changes in the thyroid, testes, and kidneys in any of the groups administered [^211^At]PSMA-5 (5, 12, and 35 MBq/kg) compared to the control group (Figure 4). Increased single-cell necrosis/apoptosis was observed in the striatal epithelial and serous cells of the salivary gland in 1/10 and 7/10 mice in the 12 MBq/kg and 35 MBq/kg groups, respectively, which was attributed to [^211^At]PSMA-5 administration (Figure 4 and Supplemental Table S5-1). Additionally, a dose-dependent increase in single-cell necrosis/apoptosis of crypt epithelial cells was noted in the pyloric glands of the stomach, duodenum, and jejunum. A dose-dependent increase in tingible body macrophages was observed in the white pulp of the spleen and the lymphoid follicles of the mesenteric lymph nodes, along with a decrease in myeloid cells in the bone marrow (Figure 4 and Supplemental Table S5-1). In the thymus, cortical lymphopenia was noted in the highest-dose group (2/10 at 35 MBq/kg), suggesting a possible relationship with the decrease in bone marrow cells (9/10 at 35 MBq/kg) (Figure 4). Degenerative changes in the salivary glands, stomach, duodenum, jejunum, spleen, mesenteric lymph nodes, bone marrow, and thymus were not observed on day 14 post-administration of [^211^At]PSMA-5, indicating its reversibility (Supplemental Table S5-2). No new histopathological findings were observed on day 14.

Mild localized hepatocellular necrosis was observed in the liver of one mouse (1/10) in the 35 MBq/kg group, which was considered to be a spontaneously occurring lesion in mice. Similarly, atrophy of the seminal vesicles (1/10) and an intraperitoneal mass (1/10) in the 12 MBq/kg group were also regarded as spontaneous lesions by the expert evaluation. Submucosal hemorrhage in the bladder was also observed in the control group, which was attributed to the urine collection procedure (Figure 4 and Supplemental Table S5-1). In the stomach, in addition to necrosis/apoptosis, gastric gland dilatation and inflammatory cell infiltration were observed in autopsies on days 1 and 14 post-administration. These changes were not dose-related and were also observed in the control group, which were deemed to be naturally occurring lesions. No abnormalities were found in the histological analyses of the other organs.

**Figure 5:**
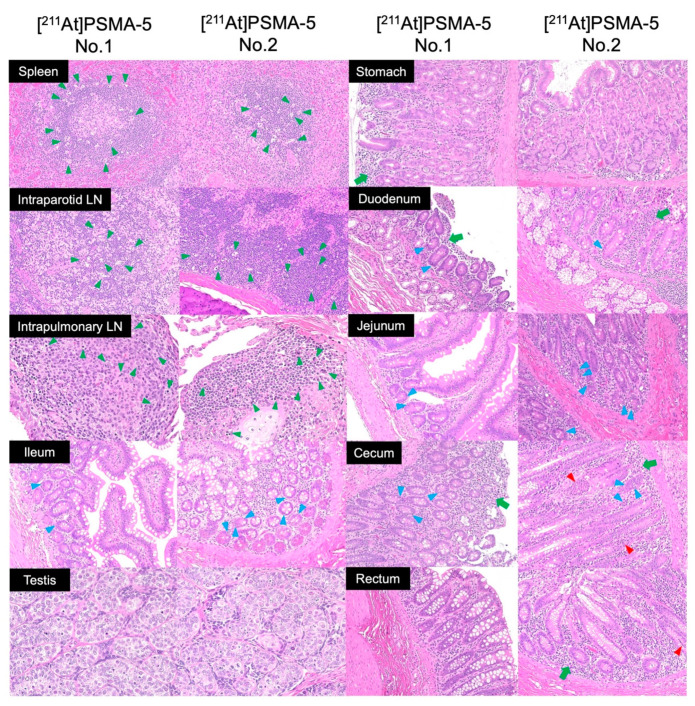
HE-stained section of cynomolgus monkey (day 1) after administration of [^211^At]PSMA-5 (9 MBq/kg) (magnification ×200: other than testes). Tingible body macrophages (green arrow heads) are observed in the spleen and intraparotid and intrapulmonary lymph nodes (LNs). Single-cell necrosis/apoptosis of crypt epithelial cells (blue arrow heads) are observed in the duodenum, jejunum, ileum, and cecum. Mild mononuclear cell infiltration of lymphocytes and plasma cells (green arrows) was observed in the lamina propria. A crypt abscess filled with inflammatory cells (red arrow heads) is observed in the duct. These inflammatory changes are spontaneous lesions. Testes (magnification ×400) show no cavity formation within the seminiferous tubules, indicating the histological structure of immature seminiferous tubules without spermatogenesis, compatible with sexually immature animals.

Histological results of the monkeys are shown in Figure 5 and Supplemental Table S5-3. A mild increase in the number of tingible body macrophages was observed in the white pulp of the spleen, intraparotid lymph nodes, and intrapulmonary lymph nodes. Mild single-cell necrosis/apoptosis of crypt epithelial cells was observed in the small and large (cecum) intestines. Spontaneous inflammatory changes were observed in the mucosa of the stomach, duodenum, cecum, and rectum. No pathological abnormalities related to the administration of [^211^At]PSMA-5 were observed in the brain, salivary glands, thyroid, heart, liver, gallbladder, pancreas, rectum, kidney, bladder, adrenal glands, or prostate.

## 3. Discussion

In this study, we evaluated the biodistribution and preclinical toxicity of [^211^At]PSMA-5 in mice and monkeys according to the ICH M3 (R2) and S9 guidelines, laying the groundwork for its use in a first-in-human clinical trial. No severe toxicity or death was observed in mice at the highest dose of 35 MBq/kg during the 14-day observation period. According to the ICH S9 guidelines, the starting dose in humans is typically set at 1/10 of the dose that causes severe toxicity in 10% of test animals (STD10) in rodents. Based on these findings, we determined that the STD10 for [^211^At]PSMA-5 will exceed 35 MBq/kg in mice (2.8 MBq/kg in human after corrected by body surface area conversion) [25]. This dose is used as a reference for setting the starting dose in the forthcoming clinical trial.

High accumulation of [^211^At]PSMA-5 in the kidneys has been observed in biodistribution studies, reflecting PSMA’s physiological expression in the proximal tubules [26]. The thyroid gland exhibited moderate uptake, possibly due to the dehalogenation of free At^−^ from the compound. However, the histological evaluation revealed no abnormalities in either the kidneys or thyroid in the highest-dose group of mice (35 MBq/kg) or monkeys (9 MBq/kg). In contrast, previous toxicity studies using [^211^At]NaAt revealed thyroid ablation effects histologically, even in low-dose groups (5 MBq/kg), attributable to its high uptake in the thyroid (509 %ID/g at 3 h post-administration) compared with 51 %ID/g in [^211^At]PSMA-5 [27]. In kidneys, previous study reported the late nephropathy with loss of the proximal tubules in mice treated with 1.5MBq (75MBq/kg) of ^211^At-labeled PSMA ligand (^211^At-6) [28]. In addition, regenerated tubules were observed in the kidneys 3 and 6 weeks after administration of [^211^At]PSMA-5 (1MBq, 53MBq/kg) in LNCaP xenograft mice [6]. These reports indicated that late nephrotoxicity should be taken into consideration at high dose administration.

On day 1, dose-dependent increases in single-cell necrosis/apoptosis were evident in the salivary glands of mice and intestinal tracts of both mice and monkeys. Stem cells in the crypts, which proliferate to continuously regenerate intestinal villi and maintain tissue structure, are known to be sensitive to radiation, including α-rays [29,30]. Tingible body macrophages observed in the spleen and lymph nodes of mice and monkeys on day 1 indicated macrophage phagocytosis of B lymphocytes undergoing apoptosis induced by alpha rays [31]. In the mice group administered 35 MBq/kg, cortical lymphopenia was observed in the thymus, likely associated with a decline in myeloid cells, given that the thymus receives lymphoid progenitor cells from the bone marrow [32]. These changes were transient, with no significant toxicity observed 14 days after administration in mice.

Regarding myelosuppression, mice exhibited no significant decrease in blood cells, whereas monkeys showed mild leukopenia (lymphopenia) 24 h post-administration. In mice, a dose-dependent decrease in bone marrow cells was observed on day 1 in histological analysis; however, no significant change was observed in the number of peripheral blood cells, indicating sufficient recovery reserve by day 14.

Species differences were observed in urine excretion between mice and monkeys. While urinary excretion was confirmed in mice, monkeys showed minimal to no urine excretion with no visual accumulation of urine in their bladders. The structure of [^211^At]PSMA-5 is similar to the PSMA-PET probe [^18^F]PSMA-1007, and a similar trend was noted, possibly due to species differences in renal tubular reabsorption [33,34,35,36]. In addition, the estimated absorbed dose in salivary glands was higher in monkeys (3.51 mGy/MBq) than in mice (0.218 mGy/MBq) (Table 1 and Supplemental Table S1). High physiological accumulation in human salivary glands was noted on PSMA-PET, and caution is advised due to the potential risk of toxicity, such as xerostomia. There are also differences in the estimated absorbed doses for the heart, liver, and kidneys, which can be attributed to differences in distribution kinetic rates between mice and monkeys. Additionally, mice are measured with gamma-counter following dissection, whereas cynomolgus monkeys are measured using SPECT. Since these estimates are based on imaging, partial volume effects and other factors may have influenced the results.

We used potassium iodide (KI) as a catalyst for the radiolabeling of PSMA-5, which is a precursor molecule coupled with a boronic acid. PSMA-5 was labeled with ^211^At by a borono-astatine substitution reaction, through an intermediate molecule, [^211^At]AtI or [^211^At]AtI_2_, formed from ^211^At and KI in the reaction mixture. The radiochemical yields of the reaction were high (not less than 90%), and not affected by the amount of KI in a wide range. The mechanism of the reaction is described in detail in the previous paper [37]. The rates of conversion of PSMA-5 to Iodo-PSMA-5 during the reaction were not more than 1% (<0.06 µg in the bulk reaction mixture) in HPLC analysis. KI was completely removed from the final product by the solid-phase extraction method. We believe that neither Iodo-PSMA-5 nor KI significantly affects the pharmacokinetics of [^211^At]PSMA-5, as their quantities are negligibly small.

This study had some limitations. First, establishing groups with doses higher than 35 MBq/kg in mice or 9 MBq/kg in monkeys was challenging because of the legal limitations on facility usage and frequency of astatine supply for preclinical use at Osaka University. Second, in consideration of animal welfare, only two cynomolgus monkeys were evaluated, limiting comparisons with controls or the evaluation of late-phase recovery. Despite these challenges, preclinical data were collected from two species (rodents and monkeys) in this study in alignment with the ICH S9 guideline. Combined with the clinical experience from an ongoing clinical trial using [^211^At]NaAt (Alpha-T1 study, NCT05275946), these data will assist in determining the appropriate starting dose in humans and the dose-escalation design for the first-in-human clinical trial using [^211^At]PSMA-5.

## 4. Materials and Methods

### 4.1. Preparation of [^211^At]PSMA-5

^211^At was produced by a nuclear reaction of ^209^Bi(α, 2n)^211^At using a cyclotron and purified by a dry distillation method, resulting in an aqueous solution of ^211^At (0.1–1.0 MBq/μL). [^211^At]PSMA-5 was synthesized via the Shirakami reaction as described in our previous study (Supplemental Figure S1) [6]. Briefly, within a microtube, 1–6 µg of PSMA-5 was dissolved in a mixture containing 7% sodium bicarbonate (0.1 mL), 0.1 mol/L potassium iodide (0.1 mL), and water (0.1 mL). An aliquot of the ^211^At aqueous solution (30–100 MBq) was then introduced to the tube, followed by a reaction period at 80 ºC for 45 min. Subsequent purification was achieved using a solid-phase extraction cartridge. The labeling yield and radiochemical purity of the resulting products were both ≥90%, with a molar activity ranging between 60–100 MBq/nmol.

### 4.2. Animal Preparation and Administration of [^211^At]PSMA-5

This study was conducted in compliance with the Animal Experiment Regulations and the Act on Welfare and Management of Animals of the Osaka University Graduate School of Medicine (approval numbers: 04-070 and 04-094). Normal male ICR mice (n = 85, 5–6 weeks old, body weight: 29.4 ± 2.3 g) and cynomolgus monkey (n = 2, body weight: 2.10 and 2.05 kg) were purchased from Japan SLC Inc. (Hamamatsu, Japan) and Hamri Co., Ltd. (Ibaraki, Japan), respectively. The animals were housed under a 12 h light/12 h dark cycle. General condition and body weight were monitored.

[^211^At]PSMA-5 was administered intravenously to 25 mice, and its biodistribution was evaluated. For the toxicity study, three doses of [^211^At]PSMA-5 (5, 12, and 35 MBq/kg, single dose) or saline were intravenously administered to 10 mice/group for the main evaluation point on day 1, and 5-mice/group for recovery evaluation on day 14, adhering to the ICH M3 (R2) guidelines. The dosage was determined based on our previous study using [^211^At]NaAt [24]. The monkeys were monitored for 24 h post-administration of [^211^At]PSMA-5 (9 MBq/kg).

### 4.3. Biodistribution Study, Metabolite Study, and Estimation of Absorbed Dose in Human

Under isoflurane inhalation anesthesia, blood samples were drawn from the inferior vena cava of mice 10 min, 1 h, 3 h, 6 h, and 24 h after administration of [^211^At]PSMA-5 (5 MBq/kg: approximately 0.15 MBq/100 μL, each n = 5), followed by euthanasia. Monkeys were anesthetized with intramuscular administration of ketamine (10 mg/kg) and xylazine (0.5 mg/kg) with isoflurane inhalation anesthesia. Planar and Single Photon Emission Computed Tomography (SPECT) imaging was performed in monkeys using a γ-camera system (E-cam, Siemens, Erlangen, Germany) immediately after administration to 1 h and at 3 and 24 h post-administration of [^211^At]PSMA-5 (9 MBq/kg), targeting the X-rays emitted from the daughter nuclide ^211^Po (energy window: 79 keV ± 20%) [23,24]. Subsequent image analysis was performed by setting the regions of interest using Osirix MD software (Version 13, Pixmeo SARL, Bernex, Switzerland) and PMOD software (Version 3.903, PMOD Technologies, Fällanden, Switzerland). Blood samples were obtained pre-administration and at 1, 3, and 24 h post-administration, followed by euthanasia via bloodletting at 24 h.

Urine and feces samples were collected at each time point. Following euthanasia, the brain, thyroid gland, salivary gland, lungs, heart, liver, kidney, spleen, pancreas, stomach, small intestine, large intestine, bladder, testes, and prostate tissues were collected. Organ weights and radiation doses were measured using a γ-counter (model: 2480Wizard2, PerkinElmer) and γ-camera system (E-cam) by appropriate cross-calibration.

The blood samples were centrifuged at 3000 rpm for 5 min, and the resulting plasma was mixed with twice the volume of ethanol, then agitated vigorously. The supernatants were analyzed by thin-layer chromatography (TLC).

The residence time (h) was calculated by determining the area under the time–radioactivity curve in mice, using an exponential decay function according to the physical half-life. Subsequently, assuming a comparable whole-body distribution between mice and humans, we adjusted the residence time by incorporating organ weight corrections aligned with human reference organ weights, with reference to the International Commission on Radiological Protection (ICRP) Publication 89 [38]. This residence time was subsequently used as input for the IDAC-Dose (Version 2.1, an internal exposure dose calculation software) to determine the absorbed doses for the major organs [39]. Absorbed doses were also estimated from SPECT images in monkeys.

### 4.4. Blood Examination

A blood cell counter was used for the measurements (CB-1010; ARKRAY, Inc., Kyoto, Japan). Red blood cell count (RBC), hemoglobin concentration (Hb), hematocrit (Ht), white blood cell count (WBC), platelet count (platelet), lymphocytes (%Lymph), monocytes (%Mon), and granulocytes (%Gra) were calculated. Plasma was analyzed using a dry clinical chemistry analyzer (Spotchem D-00 QR D-02; ARKRAY, Inc., Kyoto, Japan). The levels of aspartate aminotransferase (AST), alanine aminotransferase (ALT), gamma-glutamyl transpeptidase (γ-GTP), lactate dehydrogenase (LDH), alkaline phosphatase (ALP), creatine kinase (CK), amylase (AMY), total bilirubin (TBIL), creatinine (CRE), urea nitrogen (BUN), glucose (GLU), total protein (TP), albumin (ALB), total cholesterol (TCHO), triglyceride (TG), and electrolytes (Na, K, and Cl) were measured.

### 4.5. Histological Evaluation and Organ Weight Measurement

The brain, salivary gland, thyroid, heart, lungs, liver, kidney, adrenal gland, pancreas, spleen, thymus, stomach, small intestine, large intestine, bladder, prostate, and testes were collected on days 1 or 14 in mice and at 24 h in monkeys. Additionally, the pituitary gland, tongue, trachea, esophagus, mesenteric lymph nodes, epididymis, seminal vesicles, skin, mammary glands, spinal cord, bone, bone marrow (femur), eyeball, and optic nerve were collected from the mice. After fixation, paraffin sections were prepared and stained with hematoxylin and eosin (HE). All the specimens were evaluated by an expert in toxic pathology using an integrated fluorescence microscope (BZ-X810; Keyence Corporation. Osaka, Japan). In mice, the brain, salivary glands, heart, lungs, liver, gallbladder, spleen, kidneys, and testes were weighed according to ICH S4 guideline [40].

### 4.6. Statistical Analysis

The mean values of the control group and each administration group were compared using Dunnett’s test. All statistical analyses were performed using SPSS (version 19.0). Statistical significance was set at *p* < 0.05.

## 5. Conclusions

In this meticulously conducted preclinical evaluation of [^211^At]PSMA-5, adhering to stringent reliability standards and assessed by toxicological pathology experts, no severe toxicity emerged in mice even at doses up to 35 MBq/kg. Similarly, in a cynomolgus monkey study, at a dose of 9 MBq/kg, biodistribution and histological findings comparable to those in mice were noted, although the monkeys exhibited a higher radiation-induced effects in blood count and some blood chemistry parameters. [^211^At]PSMA-5 demonstrates potential as an advanced next-generation targeted alpha therapy for prostate cancer, particularly considering its sustainable production using a cyclotron.

## Figures and Tables

**Table 1:** Estimated absorbed doses in humans from the biodistribution in mice post-administration of [^211^At]PSMA-5.

Organ	Estimated Absorbed Dose (mGy/MBq)
Brain	0.0123
Thyroid	1.82
Salivary glands	0.218
Heart	0.232
Lung	0.0478
Liver	0.0708
Stomach	0.258
Small intestine	0.089
Colon	0.087
Kidney	4.05
Pancreas	0.105
Spleen	0.242
Testes	0.087
Prostate	0.17
Bladder	0.147
Red bone marrow	0.055

**Table 2:** Hematological examination findings in mice following a single intravenous dose of [^211^At]PSMA-5 (n = 10 for day 1, n = 5 for day 14, respectively). Data are expressed as mean ± standard deviation.

(Day 1)	Control	5 MBq/kg	12 MBq/kg	35 MBq/kg
RBC (10^6^ cell/µL)	7.6 ± 0.3	7.8 ± 0.3	8.0 ± 0.5	7.9 ± 0.3
Hb (g/dL)	12.8 ± 0.7	12.9 ± 0.4	13.0 ± 0.8	12.8 ± 0.7
Ht (%)	41.8 ± 1.9	42.5 ± 1.4	42.6 ± 2.3	42.3 ± 2
WBC (10^3^ cell/µL)	8.0 ± 3.7	6.3 ± 4.1	8.3 ± 4.9	8.0 ± 5.2
Platelet (10^3^ cell/µL)	915 ± 343	976 ± 524	1329 ± 845	952 ± 444
%Lymph (%)	59.4 ± 16.4	66.3 ± 15.8	62.6 ± 14.2	53.5 ± 16.9
(Day 14)	Control	5 MBq/kg	12 MBq/kg	35 MBq/kg
RBC (10^6^ cell/µL)	8.7 ± 0.5	8.7 ± 0.2	8.3 ± 0.6	8.4 ± 0.4
Hb (g/dL)	14.2 ± 1	14.4 ± 0.4	13.8 ± 0.9	13.6 ± 0.7
Ht (%)	45.8 ± 3.1	45.4 ± 1	43.9 ± 3	43.1 ± 2.4
WBC (10^3^ cell/µL)	9.6 ± 5.4	6.9 ± 3.3	6.9 ± 1.9	5.4 ± 2.8
Platelet (10^3^ cell/µL)	777 ± 381	1120 ± 555	922 ± 608	1331 ± 1037
%Lymph (%)	63.9 ± 11.6	70 ± 8.8	59.9 ± 12.1	53.9 ± 6.4

**Table 3:** Hematological examination findings in monkeys (*n* = 2) following a single intravenous administration of [^211^At]PSMA-5.

	Pre-Administration	1 h	3 h	24 h
RBC (10^6^ cell/µL)	4.80 ± 0.10	4.30 ± 0.30	4.70 ± 0.00	4.20 ± 0.10
Hb (g/dL)	11.2 ± 0.8	10.3 ± 1.4	11.2 ± 0.8	9.9 ± 0.5
Ht (%)	35.9 ± 2.0	32.9 ± 4.8	35.4 ± 2.5	31.8 ± 1.5
WBC (10^3^ cell/µL)	14.3 ± 3.9	10.1 ± 0.7	6.7 ± 1.4	5.3 ± 0.8
Platelet (10^3^ cell/µL)	134.2 ± 56.8	263.7 ± 33.0	306.8 ± 41.2	269.7 ± 60.8
%Lymph (%)	57.0 ± 8.0	44.0 ± 16.0	52.0 ± 4.5	23.7 ± 1.1

## Data Availability

Data is available upon request.

## Supplementary Materials

The following supporting information can be downloaded at: https://www.mdpi.com/article/10.3390/ijms25115667/s1.

## References

[1] Hofman M.S., Lawrentschuk N., Francis R.J., Tang C., Vela I., Thomas P., Rutherford N., Martin J.M., Frydenberg M., Shakher R. (2020). Prostate-specific membrane antigen PET-CT in patients with high-risk prostate cancer before curative-intent surgery or radiotherapy (proPSMA): A prospective, randomised, multicentre study. Lancet.

[2] Chow K.M., So W.Z., Lee H.J., Lee A., Yap D.W.T., Takwoingi Y., Tay K.J., Tuan J., Thang S.P., Lam W. (2023). Head-to-head Comparison of the Diagnostic Accuracy of Prostate-specific Membrane Antigen Positron Emission Tomography and Conventional Imaging Modalities for Initial Staging of Intermediate- to High-risk Prostate Cancer: A Systematic Review and Meta-analysis. Eur. Urol..

[3] Mattana F., Muraglia L., Barone A., Colandrea M., Diffalah Y.S., Provera S., Cascio A.S., Salè E.O., Ceci F. (2024). Prostate-Specific Membrane Antigen-Targeted Therapy in Prostate Cancer: History, Combination Therapies, Trials, and Future Perspective. Cancers.

[4] Kratochwil C., Fendler W.P., Eiber M., Hofman M.S., Emmett L., Calais J., Osborne J.R., Iravani A., Koo P., Lindenberg L. (2023). Joint EANM/SNMMI procedure guideline for the use of 177Lu-labeled PSMA-targeted radioligand-therapy (177Lu-PSMA-RLT). Eur. J. Nucl. Med. Mol. Imaging.

[5] Hofman M.S., Emmett L., Sandhu S., Iravani A., Joshua A.M., Goh J.C., Pattison D.A., Tan T.H., Kirkwood I.D., Ng S. (2021). [177Lu]Lu-PSMA-617 versus cabazitaxel in patients with metastatic castration-resistant prostate cancer (TheraP): A randomised, open-label, phase 2 trial. Lancet.

[6] Keam S.J. (2022). Lutetium Lu 177 Vipivotide Tetraxetan: First Approval. Mol. Diagn. Ther..

[7] Sartor O., de Bono J., Chi K.N., Fizazi K., Herrmann K., Rahbar K., Tagawa S.T., Nordquist L.T., Vaishampayan N., El-Haddad G. (2021). Lutetium-177-PSMA-617 for metastatic castration-resistant prostate cancer. N. Engl. J. Med..

[8] Sartor A.O., Morris M.J., Chi K.N., De Bono J.S., Shore N.D., Crosby M., Kreisl T.N., Fizazi K. (2022). PSMAfore: A phase 3 study to compare ^177^Lu-PSMA-617 treatment with a change in androgen receptor pathway inhibitor in taxane-naïve patients with metastatic castration-resistant prostate cancer. J. Clin. Oncol..

[9] Kratochwil C., Bruchertseifer F., Giesel F.L., Weis M., Verburg F.A., Mottaghy F., Kopka K., Apostolidis C., Haberkorn U., Morgenstern A. (2016). 225Ac-PSMA-617 for PSMA-targeted α-radiation therapy of metastatic castration-resistant prostate cancer. J. Nucl. Med..

[10] Sathekge M.M., O Lawal I., Bal C., Bruchertseifer F., Ballal S., Cardaci G., Davis C., Eiber M., Hekimsoy T., Knoesen O. (2024). Actinium-225-PSMA radioligand therapy of metastatic castration-resistant prostate cancer (WARMTH Act): A multicentre, retrospective study. Lancet Oncol..

[11] Feuerecker B., Tauber R., Knorr K., Heck M., Beheshti A., Seidl C., Bruchertseifer F., Pickhard A., Gafita A., Kratochwil C. (2021). Activity and Adverse Events of Actinium-225-PSMA-617 in Advanced Metastatic Castration-resistant Prostate Cancer After Failure of Lutetium-177-PSMA. Eur. Urol..

[12] Zacherl M.J., Gildehaus F.J., Mittlmeier L., Böning G., Gosewisch A., Wenter V., Unterrainer M., Schmidt-Hegemann N., Belka C., Kretschmer A. (2021). First Clinical Results for PSMA-Targeted α-Therapy Using 225Ac-PSMA-I&T in Advanced-mCRPC Patients. J. Nucl. Med..

[13] Parida G.K., Panda R.A., Bishnoi K., Agrawal K. (2023). Efficacy and Safety of Actinium-225 Prostate-Specific Membrane Antigen Radioligand Therapy in Metastatic Prostate Cancer: A Systematic Review and Metanalysis. Med. Princ. Pract..

[14] Kratochwil C., Haberkorn U., Giesel F.L. (2020). 225Ac-PSMA-617 for Therapy of Prostate Cancer. Semin. Nucl. Med..

[15] Ndlovu H., Mokoala K.M.G., Lawal I., Emmett L., Sathekge M.M. (2024). Prostate-specific Membrane Antigen: Alpha-labeled Radiopharmaceuticals. PET Clin..

[16] Sathekge M., Bruchertseifer F., Knoesen O., Reyneke F., Lawal I., Lengana T., Davis C., Mahapane J., Corbett C., Vorster M. (2019). 225Ac-PSMA-617 in chemotherapy-naive patients with advanced prostate cancer: A pilot study. Eur. J. Nucl. Med. Mol. Imaging.

[17] Ostuni E., Taylor M.R.G. (2022). Commercial and business aspects of alpha radioligand therapeutics. Front. Med..

[18] Watabe T., Kaneda-Nakashima K., Liu Y., Shirakami Y., Ooe K., Toyoshima A., Shimosegawa E., Fukuda M., Shinohara A., Hatazawa J. (2019). Enhancement of ^211^At uptake via the sodium iodide symporter by the addition of ascorbic acid in targeted α-therapy of thyroid cancer. J. Nucl. Med..

[19] Zalutsky M.R., Pruszynski M. (2011). Astatine-211: Production and availability. Curr. Radiopharm..

[20] Rabiei M., Asadi M., Yousefnia H. (2023). Astatine-211 Radiopharmaceuticals; Status, Trends, and the Future. Curr. Radiopharm..

[21] Feng Y., Zalutsky M.R. (2021). Production, purification and availability of 211At: Near term steps towards global access. Nucl. Med. Biol..

[22] McIntosh L.A., Burns J.D., Tereshatov E.E., Muzzioli R., Hagel K., Jinadu N.A., McCann L.A., Picayo G.A., Pisaneschi F., Piwnica-Worms D. (2023). Production, isolation, and shipment of clinically relevant quantities of astatine-211: A simple and efficient approach to increasing supply. Nucl. Med. Biol..

[23] Watabe T., Kaneda-Nakashima K., Shirakami Y., Kadonaga Y., Ooe K., Wang Y., Haba H., Toyoshima A., Cardinale J., Giesel F.L. (2023). Targeted α-therapy using astatine (^211^At)-labeled PSMA1, 5, and 6: A preclinical evaluation as a novel compound. Eur. J. Nucl. Med. Mol. Imaging.

[24] Watabe T., Kaneda-Nakashima K., Ooe K., Liu Y., Kurimoto K., Murai T., Shidahara Y., Okuma K., Takeuchi M., Nishide M. (2021). Extended single-dose toxicity study of [^211^At]NaAt in mice for the first-in-human clinical trial of targeted alpha therapy for differentiated thyroid cancer. Ann. Nucl. Med..

[25] Nair A.B., Jacob S. (2016). A simple practice guide for dose conversion between animals and human. J. Basic Clin. Pharm..

[26] Baccala A., Sercia L., Li J., Heston W., Zhou M. (2007). Expression of prostate-specific membrane antigen in tumor-associated neovasculature of renal neoplasms. Urology.

[27] Liu Y., Watabe T., Kaneda-Nakashima K., Ooe K., Shirakami Y., Toyoshima A., Shimosegawa E., Nakano T., Shinohara A., Hatazawa J. (2020). Preclinical evaluation of radiation-induced toxicity in targeted alpha therapy using [^211^At] NaAt in mice : A Revisit. Transl. Oncol..

[28] Kiess A.P., Minn I., Vaidyanathan G., Hobbs R.F., Josefsson A., Shen C., Brummet M., Chen Y., Choi J., Koumarianou E. (2016). (2S)-2-(3-(1-Carboxy-5-(4-211At-Astatobenzamido)Pentyl)Ureido)-Pentanedioic Acid for PSMA-Targeted α-Particle Radiopharmaceutical Therapy. J. Nucl. Med..

[29] Xiao S., Zhou L. (2020). Gastric Stem Cells: Physiological and pathological perspectives. Front. Cell Dev. Biol..

[30] Andreu P., Colnot S., Godard C., Gad S., Chafey P., Niwa-Kawakita M., Laurent-Puig P., Kahn A., Robine S., Perret C. (2005). Godard1. Crypt-restricted proliferation and commitment to the Paneth cell lineage following Apc loss in the mouse intestine. Development.

[31] Grootveld A.K., Kyaw W., Panova V., Lau A.W., Ashwin E., Seuzaret G., Dhenni R., Bhattacharyya N.D., Khoo W.H., Biro M. (2023). Apoptotic cell fragments locally activate tingible body macrophages in the germinal center. Cell.

[32] Zlotoff D.A., Bhandoola A. (2011). Hematopoietic progenitor migration to the adult thymus. Ann. N. Y. Acad. Sci..

[33] Watabe T., Uemura M., Soeda F., Naka S., Ujike T., Hatano K., Sasaki H., Kamiya T., Shimosegawa E., Kato H. (2021). High detection rate in [^18^F]PSMA-1007 PET: Interim results focusing on biochemical recurrence in prostate cancer patients. Ann. Nucl. Med..

[34] Giesel F.L., Hadaschik B., Cardinale J., Radtke J., Vinsensia M., Lehnert W., Kesch C., Tolstov Y., Singer S., Grabe N. (2017). F-18 labelled PSMA-1007: Biodistribution, radiation dosimetry and histopathological validation of tumor lesions in prostate cancer patients. Eur. J. Nucl. Med. Mol. Imaging.

[35] Sprute K., Kramer V., Koerber S.A., Meneses M., Fernandez R., Soza-Ried C., Eiber M., Weber W.A., Rauscher I., Rahbar K. (2021). Diagnostic Accuracy of 18F-PSMA-1007 PET/CT Imaging for Lymph Node Staging of Prostate Carcinoma in Primary and Biochemical Recurrence. J. Nucl. Med..

[36] Giesel F.L., Knorr K., Spohn F., Will L., Maurer T., Flechsig P., Neels O., Schiller K., Amaral H., Weber W.A. (2019). Detection Efficacy of 18F-PSMA-1007 PET/CT in 251 Patients with Biochemical Recurrence of Prostate Cancer After Radical Prostatectomy. J. Nucl. Med..

[37] Shirakami Y., Watabe T., Obata H., Kaneda K., Ooe K., Liu Y., Teramoto T., Toyoshima A., Shinohara A., Shimosegawa E. (2021). Synthesis of [^211^At]4-astato-L-phenylalanine by dihydroxyboryl-astatine substitution reaction in aqueous solution. Sci. Rep..

[38] Valentin J. (2002). Basic anatomical and physiological data for use in radiological protection: Reference values: ICRP Publication 89: Approved by the Commission in September 2001. Ann. ICRP.

[39] Andersson M., Johansson L., Eckerman K., Mattsson S. (2017). IDAC-Dose 2.1, an internal dosimetry program for diagnostic nuclear medicine based on the ICRP adult reference voxel phantoms. EJNMMI Res..

[40] (1998). ICH Harmonized Tripartite Guideline: Duration of Chronic Toxicity Testing in Animals (Rodent and Non Rodent Toxicity Testing) S4. International Conference on Harmonisation of Technical Requirements for Registration of Pharmaceuticals for Human Use. https://database.ich.org/sites/default/files/S4_Guideline.pdf.

