# Supplementary material for: Preclinical Evaluation of Biodistribution and Toxicity of [211At]PSMA-5 in Mice and Primates for the Targeted Alpha Therapy against Prostate Cancer
Source: Int J Mol Sci. 2024 May 23;25(11):5667. doi: 10.3390/ijms25115667 (PMC11172375; doi:10.3390/ijms25115667)
Supplement: Supplementary file 1 [file ijms-25-05667-s001.zip › ijms-2993023-supplementary/Supplemental Figure S1.pdf]

[^{211}At]PSMA-5

(((S)-5-((R)-2-(4-(((R)-2-((R)-2-(2-(4-astatophenyl)acetamido)-4-carboxybutanamido)-4-carboxybutanamido)methyl)benzamido)-3-(naphthalen-2-yl)propanamido)-1-carboxypentyl)carbamoyl)-L-glutamic acid

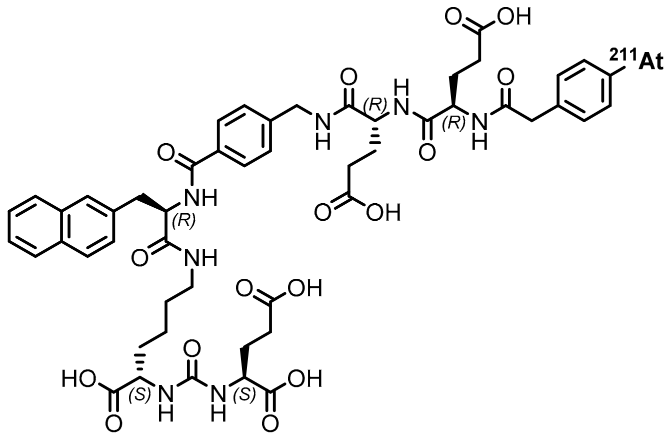

PSMA5,

(((S)-5-((R)-2-(4-(((R)-2-((R)-2-(2-(4-boronophenyl)acetamido)-4-carboxybutanamido)-4-carboxybutanamido)methyl)benzamido)-3-(naphthalen-2-yl)propanamido)-1-carboxypentyl)carbamoyl)-L-glutamic acid,

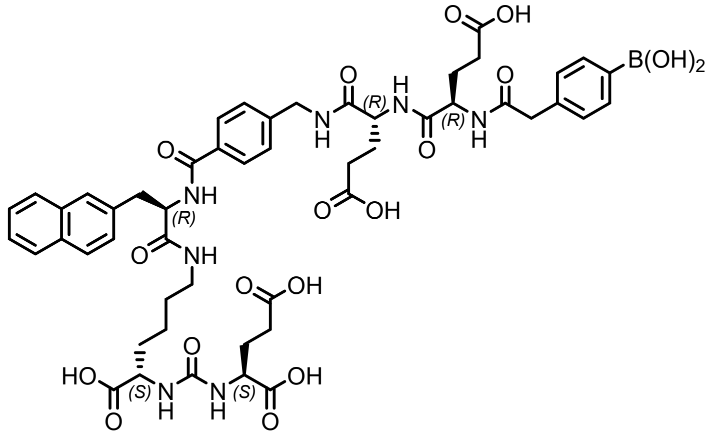

Supplemental Figure S1. Chemical names and structures of [^{211}At]PSMA-5 and PSMA-5 precursors
