# Supplementary material for: Preclinical Evaluation of Biodistribution and Toxicity of [211At]PSMA-5 in Mice and Primates for the Targeted Alpha Therapy against Prostate Cancer
Source: Int J Mol Sci. 2024 May 23;25(11):5667. doi: 10.3390/ijms25115667 (PMC11172375; doi:10.3390/ijms25115667)
Supplement: Supplementary file 1 [file ijms-25-05667-s001.zip › ijms-2993023-supplementary/Supplemental Table_S1.pdf]

Supplemental Table S1. Estimated absorbed doses in humans from the biodistribution in monkeys for organs which were identified on SPECT images at 1,3,24 hrs post-administration of [^{211}At]PSMA-5.

Organ	Estimated absorbed dose from monkey (mGy/MBq)
Salivary glands	3.51
Heart	0.93
Liver	0.25
Kidney	1.41
