# Supplementary material for: Preclinical Evaluation of Biodistribution and Toxicity of [211At]PSMA-5 in Mice and Primates for the Targeted Alpha Therapy against Prostate Cancer
Source: Int J Mol Sci. 2024 May 23;25(11):5667. doi: 10.3390/ijms25115667 (PMC11172375; doi:10.3390/ijms25115667)
Supplement: Supplementary file 1 [file ijms-25-05667-s001.zip › ijms-2993023-supplementary/Supplemental Tables_S2-S3.pdf]

Supplemental Table S2. Results of the blood chemistry examination in mice following a single intravenous administration of [^{211}At]PSMA-5 (n=10 for day 1, n=5 for day 14, respectively).

(Day1)	Control	5 MBq/kg	12 MBq/kg	35 MBq/kg
AST (IU/L)	38.6 ± 15.6	43.8 ± 30.3	37 ± 11.5	51.5 ± 36.2*
ALT (IU/L)	28.5 ± 8.1	41.2 ± 31.6	29.0 ± 5.8	53.0 ± 10.3
γ-GTP (IU/L)	10 ± 0	10 ± 0	10 ± 0	10 ± 0
LDH (IU/L)	573 ± 141	641 ± 281	736 ± 363	638 ± 385
ALP (IU/L)	273 ± 164	194 ± 86	193 ± 100	208 ± 67
CK (IU/L)	164 ± 59	180 ± 60	192 ± 50	170 ± 65
AMY (IU/L)	2775 ± 658	2675 ± 562	2666 ± 537	2795 ± 363
TBIL (mg/dL)	0.61 ± 0.26	0.6 ± 0.22	0.53 ± 0.16	0.71 ± 0.48
CRE (mg/dL)	0.2 ± 0	0.2 ± 0	0.2 ± 0	0.2 ± 0
BUN (mg/dL)	22.8 ± 5.2	22 ± 8.3	20.7 ± 8.3	19.6 ± 6.2
GLU (mg/dL)	193 ± 102	198 ± 70	185 ± 42	201 ± 31
TP (g/dL)	3.9 ± 0.4	4.1 ± 0.4	4.1 ± 0.3	4.0 ± 0.3
ALB (g/dL)	2.3 ± 0.2	2.3 ± 0.2	2.3 ± 0.2	2.2 ± 0.1
TCHO (mg/dL)	121 ± 21	130 ± 20	128 ± 20	129 ± 21
TG (mg/dL)	113 ± 78	112 ± 75	119 ± 75	122 ± 79
Na (mEq/L)	140 ± 3.5	141 ± 2.5	142 ± 2.0	140 ± 1.4
K (mEq/L)	4.7 ± 0.5	4.5 ± 0.5	4.5 ± 0.3	5.1 ± 0.5
Cl (mEq/L)	115 ± 4.5	115 ± 2.8	116 ± 4.7	115 ± 4.7

(Day14)	Control	5 MBq/kg	12 MBq/kg	35 MBq/kg
AST (IU/L)	35.4 ± 12.8	45 ± 22.7	28.8 ± 15.4	28.4 ± 9.9
ALT (IU/L)	23.0 ± 8.0	16.5 ± 4.9	21.5 ± 0.7	13.0 ± 0.0
γ-GTP (IU/L)	10 ± 0	10 ± 0	10 ± 0	10 ± 0
LDH (IU/L)	689 ± 332	611 ± 24	456 ± 72	538 ± 129
ALP (IU/L)	113 ± 15	171 ± 36	110 ± 59	133 ± 46

CK (IU/L)	141 ± 34	162 ± 91	144 ± 26	141 ± 18
AMY (IU/L)	2624 ± 285	2200 ± 428	2514 ± 549	2678 ± 388
TBIL (mg/dL)	0.54 ± 0.17	0.88 ± 0.16	0.68 ± 0.13	0.68 ± 0.33
CRE (mg/dL)	0.2 ± 0	0.2 ± 0	0.22 ± 0.04	0.2 ± 0
BUN (mg/dL)	26.2 ± 3.4	27.6 ± 1.1	30.6 ± 6.1	23.4 ± 3.4
GLU (mg/dL)	182 ± 36	146 ± 39	148 ± 24	141 ± 21
TP (g/dL)	4.1 ± 0.1	4.1 ± 0.5	3.9 ± 0.4	4.1 ± 0.2
ALB (g/dL)	2.2 ± 0.1	2.2 ± 0.1	2.1 ± 0.3	2.3 ± 0.1
TCHO (mg/dL)	141 ± 15	151 ± 18	138 ± 20	144 ± 15
TG (mg/dL)	88 ± 28	75 ± 17	87 ± 42	67 ± 18
Na (mEq/L)	141 ± 1.1	143 ± 1.9	143 ± 2.8	143 ± 2.6
K (mEq/L)	5.7 ± 1.1	5.1 ± 1.2	5.4 ± 0.8	4.7 ± 0.6
Cl (mEq/L)	119 ± 2.7	121 ± 0.9	120 ± 2.9	117 ± 2.9

-: Below the limit of quantitation, *: hemolysis was observed

Supplemental Table S3. Blood chemistry analysis in monkeys (n=2) following a single intravenous administration of [^{211}At]PSMA-5 (day 1 and 14).

	Pre-administration	1 hr	3 hrs	24 hrs
AST (IU/L)	13.5 ± 3.5	13.3 ± 4.6	14 ± 5.7	38.7 ± 19.3
ALT (IU/L)	12.5 ± 3.5	11.3 ± 1.8	12 ± 2.8	24.8 ± 21
γ-GTP (IU/L)	79.5 ± 20.5	61.8 ± 8.1	66.5 ± 19.1	65 ± 17.9
LDH (IU/L)	289 ± 24.7	231 ± 27.9	255 ± 4.9	471 ± 30.2
ALP (IU/L)	932 ± 63.6	835 ± 173.6	890 ± 47.4	768 ± 34.4
CK (IU/L)	491 ± 130.1	496 ± 321.4	522 ± 101.8	713 ± 293.9
AMY (IU/L)	385 ± 79.9	291 ± 62.2	342 ± 38.2	565 ± 197
TBIL (mg/dL)	0.5 ± 0.14	0.3 ± 0.1	0.3 ± 0.0	0.7 ± 0.3
CRE (mg/dL)	0.4 ± 0.0	0.5 ± 0.2	0.75 ± 0.21	0.7 ± 0.0
BUN (mg/dL)	23 ± 1.4	24.8 ± 2.5	27 ± 1.4	35.7 ± 2.4
GLU (mg/dL)	81 ± 15.6	309 ± 177	62 ± 56.6	77.7 ± 1.4
TP (g/dL)	4.8 ± 0.3	3.6 ± 0.4	3.7 ± 0.1	4.2 ± 0.4
ALB (g/dL)	3.2 ± 0.1	2.5 ± 0.4	2.5 ± 0.1	2.7 ± 0
TCHO (mg/dL)	103 ± 7.1	84 ± 24	91 ± 17	90.7 ± 0.5
TG (mg/dL)	26.5 ± 2.1	25.5 ± 0.7	28 ± 4.2	28.7 ± 4.7
Na (mEq/L)	142 ± 2.1	140 ± 2.8	143 ± 0	146 ± 3.1
K (mEq/L)	3.6 ± 0.1	4.3 ± 0.2	4.9 ± 0.4	4 ± 0.2
Cl (mEq/L)	111 ± 3.54	104 ± 1.8	109 ± 0.71	114 ± 2.4

Relatively high AST and LDH might be affected by hemolysis. High blood glucose level at 1hr post-administration suggests contamination from the infusion fluid.
