# Supplementary material for: Preclinical Evaluation of Biodistribution and Toxicity of [211At]PSMA-5 in Mice and Primates for the Targeted Alpha Therapy against Prostate Cancer
Source: Int J Mol Sci. 2024 May 23;25(11):5667. doi: 10.3390/ijms25115667 (PMC11172375; doi:10.3390/ijms25115667)
Supplement: Supplementary file 1 [file ijms-25-05667-s001.zip › ijms-2993023-supplementary/Supplemental Tables_S4-S5.pdf]

Supplemental Table S4. Necropsy observations in mice following a single intravenous administration of [^{211}At]PSMA-5 (day 1 and 14).

Inspection time		1 day after administration			
Group		Control	5 MBq/kg	12 MBq/kg	35 MBq/kg
No. of animals		10	10	10	10
No. of surviving animals		10	10	10	10
No. of dead animals		0	0	0	0
No. of no abnormality		8	10	8	7
Organ	Necropsy findings				
Salivary gland	: Small, left	1*	0	0	0
Urinary bladder	: Red	0	0	0	2*
Kidney	: Pale	0	0	0	1*
Adrenal	: None, right	1*	0	0	0
Seminal vesicle	: Small	0	0	1*	0
Abdominal cavity	: Nodule in the ligament (dark red, 1 mm in diameter)	0	0	1*	0

Inspection time		14 days after administration			
Group		Control	5 MBq/kg	12 MBq/kg	35 MBq/kg
No. of animals		5	5	5	5
No. of surviving animals		5	5	5	5
No. of dead animals		0	0	0	0
No. of no abnormality		4	5	5	4
Organ	Necropsy findings				
Thyroid	: White, left	0	0	0	1*
Testis	: Small, left	1*	0	0	0

*: All of these changes occurred in a small number of cases and are determined to be spontaneous lesions in the histopathological examination.

Supplemental Table S5-1. Histopathological findings in mice (day 1).

Inspection time		1 day after administration																							
Group		Control						5 MBq/kg						12 MBq/kg						35 MBq/kg					
No. of animals		10						10						10						10					
Grade of lesion		N	±	+	++	+++	P	N	±	+	++	+++	P	N	±	+	++	+++	P	N	±	+	++	+++	P
Organ	Histopathological findings																								
Submandibular gland	: Single cell necrosis/apoptosis	10	0	0	0	0	0	10	0	0	0	0	0	9	1	0	0	0	0	3	2	5	0	0	0
Urinary bladder	: Hemorrhage, subepithelial, focal	4	5	1	0	0	0	Not examined						Not examined						5	3	2	0	0	0
Liver	: Necrotic foci, focal	10	0	0	0	0	0	Not examined						Not examined						9	0	1	0	0	0
Stomach	: Single cell necrosis/apoptosis, pyloric gland	10	0	0	0	0	0	10	0	0	0	0	0	10	0	0	0	0	0	8	2	0	0	0	0
	: Dilatation, gland	9	0	1	0	0	0	9	0	1	0	0	0	9	0	1	0	0	0	10	0	0	0	0	0
	: Inflammatory cell infiltration, focal	9	0	1	0	0	0	10	0	0	0	0	0	10	0	0	0	0	0	10	0	0	0	0	0
Duodenum	: Single cell necrosis/apoptosis, crypt	10	0	0	0	0	0	8	2	0	0	0	0	2	6	2	0	0	0	0	1	9	0	0	0
Jejunum	: Single cell necrosis/apoptosis, crypt	10	0	0	0	0	0	2	2	6	0	0	0	0	0	9	1	0	0	0	0	4	3	3	0
Spleen	: Tingible body macrophage, increase, white pulp	10	0	0	0	0	0	6	3	1	0	0	0	4	2	4	0	0	0	2	2	4	2	0	0
	: Extramedullary hematopoiesis	8	0	2	0	0	0	10	0	0	0	0	0	9	0	1	0	0	0	10	0	0	0	0	0
Thymus	: Cellularity decreased, lymphocyte, cortex	9	0	1	0	0	0	9	0	1	0	0	0	9	0	1	0	0	0	7	1	2	0	0	0
Mesenteric lymph node	: Tingible body macrophage, increase, lymphoid follicle	10	0	0	0	0	0	7	3	0	0	0	0	4	4	2	0	0	0	3	2	5	0	0	0
	: Cellularity decreased, lymphocyte	8	1	1	0	0	0	10	0	0	0	0	0	8	0	2	0	0	0	0	2	8	0	0	0
Bone marrow (femur)	: Cellularity decreased	10	0	0	0	0	0	5	4	1	0	0	0	0	7	3	0	0	0	0	1	6	3	0	0

Grade of lesion: N, Normal; ±, Minimal or abnormality; +, Slight; ++, Moderate; +++, Severe; P, Present.

No remarkable changes are seen in the brain, thyroid, parathyroid, trachea, esophagus, sublingual gland, heart, lung, bronchia, gallbladder, pancreas, colon, kidney, adrenal, femur, eye, and testes.

Supplemental Table S5-2. Histopathological findings in mice (day 14).

Inspection time		14 days after administration																							
Group		Control						5 MBq/kg						12 MBq/kg						35 MBq/kg					
No. of animals		5						5						5						5					
Grade of lesion		N	±	+	++	+++	P	N	±	+	++	+++	P	N	±	+	++	+++	P	N	±	+	++	+++	P
Organ	Histopathological findings																								
Urinary bladder	: Hemorrhage, subepithelium, focal	3	2	0	0	0	0	Not examined						Not examined						5	0	0	0	0	0
Liver	: Microgranuloma, focal	4	0	1	0	0	0													5	0	0	0	0	0
Stomach	: Dilatation, gland	4	0	1	0	0	0													5	0	0	0	0	0
	: Inflammatory cell infiltration, focal	5	0	0	0	0	0													4	0	1	0	0	0
Spleen	: Extramedullary hematopoiesis	4	0	1	0	0	0													5	0	0	0	0	0
Mesenteric lymph node	: Cellularity decreased, lymphocyte, cortex	2	3	0	0	0	0													3	2	0	0	0	0
Eye	: Corneal edemaa, subeptithelial, focal	4	0	1	0	0	0													5	0	0	0	0	0

Grade of lesion : N ; Normal, ± ; Minimal or abnormality, + ; Slight, ++ ; Moderate, +++ ; Severe, P ; present

No remarkable changes are seen in the brain, thyroid, parathyroid, trachea, esophagus, sublingual gland, heart, lung, bronchia, gallbladder, pancreas, duodenum, jejunum, colon, kidney, thymus, adrenal, testes, femur, and bone.

Supplemental Table S5-3. Histopathological findings in monkeys (day 1).

	Animal ID number	No.1	No.2
Organ	Histopathological findings		
Spleen		+	+
White pulp	: tingible body macrophage, increase		
Parotid gland			
Intraparotid lymph nodes	: tingible body macrophage, increase, lymphoid follicle	+	+
Lung			
Intrapulmonary lymph nodes	: tingible body macrophage, increase, lymphoid follicle	+	+
Stomach			
fundic gland	: mononuclear cell infiltration, lamina propria	+	−
pyloric gland	: mononuclear cell infiltration, lamina propria	+	−
Duodenum	: single cell necrosis/apoptosis, crypt	±	+
	: mononuclear cell infiltration, lamina propria	+	+
Jejunum	: single cell necrosis/apoptosis, crypt	+	+
Ileum	: single cell necrosis/apoptosis, crypt	+	++
Cecum	: single cell necrosis/apoptosis, crypt	+	+
	: mononuclear cell infiltration, lamina propria	+	+
	: crypt abscess	−	+
Rectum	: mononuclear cell infiltration, lamina propria	−	+
	: crypt abscess	−	+
Testis	: immature	P	P

Grade of lesion : −, None; ±, Minimal or abnormality ; +, Slight; ++, Moderate ; P, present.

No remarkable changes are seen in the brain, submandibular gland, sublingual gland, thyroid, heart, liver, gallbladder, pancreas, rectum, kidney, bladder, adrenal gland, and prostate.
